# Poultry Market Closures and Human Infection with Influenza A(H7N9) Virus, China, 2013–14

**DOI:** 10.3201/eid2011.140556

**Published:** 2014-11

**Authors:** Peng Wu, Hui Jiang, Joseph T. Wu, Enfu Chen, Jianfeng He, Hang Zhou, Lan Wei, Juan Yang, Bingyi Yang, Ying Qin, Vicky J. Fang, Ming Li, Tim K. Tsang, Jiandong Zheng, Eric H. Y. Lau, Yu Cao, Chengliang Chai, Haojie Zhong, Zhongjie Li, Gabriel M. Leung, Luzhao Feng, George F. Gao, Benjamin J. Cowling, Hongjie Yu

**Affiliations:** School of Public Health, Li Ka Shing Faculty of Medicine, The University of Hong Kong, Hong Kong Special Administrative Region, China (P. Wu, J.T. Wu, L. Wei, B. Yang, V.J. Fang, T.K. Tsang, E.H.Y. Lau, G.M. Leung, B.J. Cowling);; Division of Infectious Disease, Key Laboratory of Surveillance and Early-warning on Infectious Disease, Chinese Center for Disease Control and Prevention, Beijing, China (H. Jiang, H. Zhou, J. Yang, Y. Qin, M. Li, J. Zheng, Y. Cao, Z. Li, L. Feng, H. Yu);; Zhejiang Provincial Centre for Disease Control and Prevention, Hangzhou, China (E. Chen, C. Chai);; Guangdong Provincial Centre for Disease Control and Prevention, Guangzhou, China (J. He, H. Zhong);; CAS Key Laboratory of Pathogenic Microbiology and Immunology, Institute of Microbiology, Chinese Academy of Sciences, Beijing (G.F. Gao);; Office of Director-General, Chinese Center for Disease Control and Prevention, Beijing (G.F. Gao)

**Keywords:** Avian influenza A(H7N9), viruses, live poultry markets, public health, China

## Abstract

Closure of live poultry markets was implemented in areas affected by the influenza virus A(H7N9) outbreak in China during winter, 2013–14. Our analysis showed that closing live poultry markets in the most affected cities of Guangdong and Zhejiang provinces was highly effective in reducing the risk for H7N9 infection in humans.

A novel avian influenza A(H7N9) virus was first identified in China during March 2013, and by March 25, 2014, it had caused 390 laboratory-confirmed human cases in mainland China. The majority of patients with laboratory-confirmed cases reported recent exposure to live poultry markets (LPMs) in urban areas (*1*,*2*), and the H7N9 virus has been identified in LPMs in affected areas (*3*–*6*). Temporary closure of LPMs in Shanghai, Nangjing, Hanghzou, and Huzhou during the 2013 spring outbreak of influenza was associated with immediate and substantial reductions in incidence of confirmed H7N9 infection in those cities (*7*).

Although few confirmed human H7N9 infections were identified in the summer and autumn of 2013, the virus reemerged during the winter of 2013–14, and 251 confirmed cases were reported during December 1, 2013–March 25, 2014, mostly from cities in Guangdong Province in southern China and cities in Zhejiang Province in eastern China. In response to the identification of H7N9 in humans, poultry, or the environment, local authorities of affected cities implemented various control measures, the highest profile of which was closure of LPMs. The objective of our study was to estimate the effect of closure of LPMs in reducing incidence of human infections with H7N9 in the most affected cities of Guangdong and Zhejiang provinces during the 2013–14 winter outbreak.

## The Study

**Figure 1:**
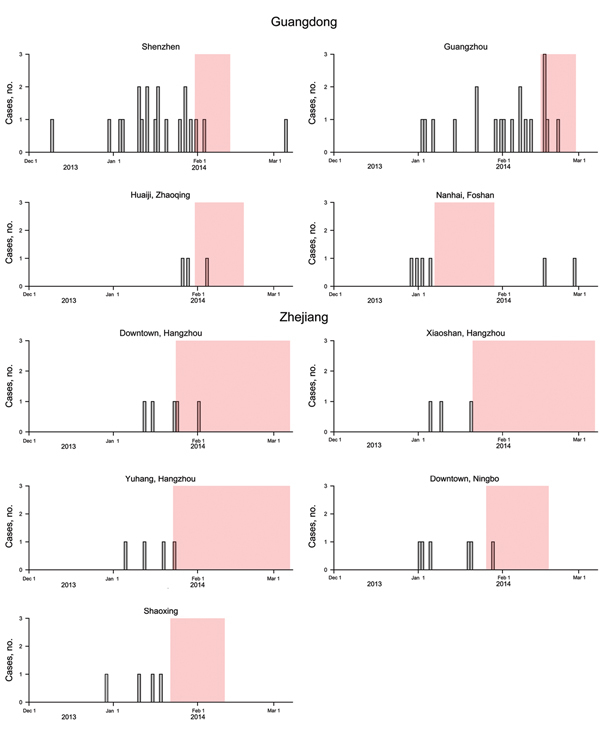
Incidence of human cases of laboratory-confirmed influenza A(H7N9) virus infection detected in urban areas of Guangdong and Zhejiang provinces, by date of illness onset, December 1, 2013–March 7, 2014. The shaded areas in each panel indicate the periods of closure of live poultry markets.

During December 1–March 25, 2014, a total of 93 and 89 confirmed human H7N9 cases were reported in Guangdong and Zhejiang provinces, respectively. In response to local cases, many urban areas introduced LPM closures for varying durations. To estimate the effect of these interventions, we used the same methods applied to estimate the effect of LPM closures in cities in eastern China in April 2013 (*7*). We selected 9 specific areas where LPMs were closed for ≥7 consecutive days, ≥3 H7N9 cases were reported in that local urban area during the study period of December 1, 2013–March 7, 2014, and >1 case was confirmed before the local LPM closure (Table). The dates of these interventions in relation to the dates of onset of illness of the 69 confirmed human cases in these 9 areas are shown in Figure 1.

**Table:** Summary of closures of live poultry markets in the urban areas of Guangdong and Zhejiang Provinces, China, during the 2013–14 winter outbreak of influenza A(H7N9)

Area	No. urban cases	Start date of LPM closure, 2014	End date of LPM closure, 2014
Guangdong Province			
Shenzhen	19	Jan 31	Feb 13
Guangzhou	19	Feb 15	Feb 28
Huaiji County, Zhaoqing	3	Jan 31 or Feb 5*	Feb 18
Nanhai District, Foshan	6	Jan 7 or Jan 13†	Jan 29
Zhejiang Province			
Hangzhou	5	Jan 24‡	Mar 7
Downtown§	3	Jan 21	Mar 7
Xiaoshan	4	Jan 23	Mar 7
Yuhang			
Ningbo downtown (4 districts)¶	6	Jan 26	Feb 18
Shaoxing	4	Jan 22	Feb 11

*Only the live poultry markets (LPMs) epidemiologically linked to cases were closed Jan 31–Feb 2, 2014. All LPMs in the county were closed Feb 5–18. †2 LPMs epidemiologically linked to cases were closed during Jan. 7–9 All LPMs in the district were closed during Jan 13–29. ‡End dates of LPM closure in these districts/cities are later than the end date of the study time horizon. §Hangzhou includes 6 districts in the downtown area (Gongshu, Shangcheng, Xiacheng, Jianggan, Xihu and Binjiang), 2 suburban districts (Xiaoshan and Yuhang), 3 cities (Fuyang, Jiande and Linan), and 2 counties (Tonglu and Chunan). ¶Ningbo includes 6 districts in the downtown area (Haishu, Jiangdong, Jiangbei, Beilun, Zhenhai , Yinzhou), 3 cities (Yuyao, Cixi , Fenghua), and 2 counties (Xiangshan, Ninghai). Four districts included here are Haishu, Jiangdong, Jiangbei, and Yinzhou.

In our analysis we assumed the following: 1) the incidence rate of human infection with H7N9 was constant for the 2 weeks before the implementation of LPM closure in each area; 2) the incidence rate of human infection with H7N9 during the LPM closure period had a different rate from the pre-closure period and was also constant, so the ratio of incidence rates during closure versus before closure indicates the effect of LPM closure, and an incidence rate <1 indicated a reduction in incidence; and 3) illness onset in each human case-patient occurred after an incubation period based on a stochastic incubation period distribution. We further assumed that the incubation periods of cases among humans in all cities followed the same lognormal distribution. The start date of the study time horizon for a given area was either 14 days before the start date of LPM closure or the onset date of the first confirmed local H7N9 case in 2014, whichever was later. The end date of the study time horizon was either the last day of local LPM closure or March 7, 2014, whichever was earlier, to allow for a possible delay of case notification for 2–3 weeks.

Our model therefore included these parameters: the incidence rates before and after closure in each city and the parameters of the incubation period distribution. We estimated these parameters using Markov chain Monte Carlo method (Technical Appendix), using flat priors for the logarithms of the incidence rate before closure and the incidence rate ratio, a lognormal distribution with mean of 3.3 days and 97.5th percentile 5.7 days for the mean incubation period, and a lognormal distribution with mean 0.76 and 97.5th percentile 5.2 for the coefficient of variation of the incubation period corresponding to the posteriors from our previous analysis (*7*). After estimating separate effects of LPM closure in each urban area, we fitted an overall model, assuming the incidence rate ratio was the same across all areas.

**Figure 2:**
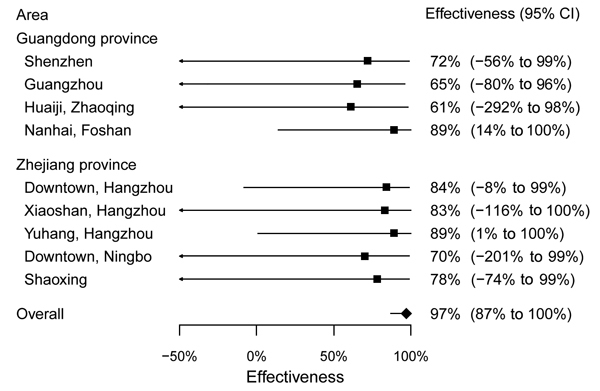
Estimates of the effect of interventions in reducing human risk for infection with avian influenza A(H7N9) virus in urban areas of Guangdong and Zhejiang provinces. Estimates are presented as effectiveness, calculated as 1 minus the ratio of incidence rates of infection after closure versus before closure, within 95% confidence intervals. Estimates are shown for each urban area, and a single summary measure is also shown assuming the effectiveness was the same across all areas. For Huaiji County and Nanhai District, live poultry markets (LPMs) epidemiologically linked to confirmed H7N9 cases were closed a few days before all the other local LPMs were closed. To account for differential start dates of LPM closure within these areas, we performed sensitivity analysis by setting the overall start date of LPM closure in a given area to be either the earliest or the last day on which local LPMs began to close. Results in the 2 scenarios were similar.

Point estimates of the effectiveness of LPM closure varied from 61%–89% in the 4 areas in Guangdong Province, and from 70%– 89% in the 5 areas in Zhejiang Province with generally wide CIs, and the effectiveness was estimated to be 97% (95% CI: 89%, 100%) in the overall model, assuming the same incidence rate ratio associated with LPM closure in each area (Figure 2). In the latter model, the incubation period distribution had a mean of 3.4 days (95% CI: 2.2–5.0) and 95th percentile of 4.8 days.

As in our previous analysis of LPM closures in April 2013 (*7*), here we found that LPM closures were effective in reducing human risk for H7N9 infection in cities in Guangdong and Zhejiang provinces during the 2013–14 winter outbreak. The relatively short closure periods and the small number of cases in each city prohibited area-specific estimates of effectiveness that had narrow credibility intervals (Figure 2). In addition, the effect of LPM closure estimated here may incorporate other contemporaneous interventions as well as potential reductions in poultry consumption and population exposure to live poultry associated with the H7N9 outbreak (*8*), seasonal variation of avian influenza cases as observed in H5N1 infections, decline in media coverage, decline in seeking of health care by possible patients, and decreased laboratory testing output related to staff fatigue during the second wave of the epidemic.

Although our results support the effectiveness of LPM closure in protecting human health, closure of LPMs is a temporary and drastic measure that may be associated with substantial costs to society and the poultry industry (*9*). More sustainable interventions are needed. In the special administrative region of Hong Kong, LPM rest days, on which stalls are cleared of unsold poultry and disinfected, have been used since 2001 to reduce the amplification of avian influenza viruses in LPMs (*10*,*11*). Ideally, improved surveillance of avian viruses in poultry would enhance identification and closure of contaminated markets and uncontaminated markets could remain open.

Our findings are limited by the ecologic nature of our analysis. It is possible that the incidence rate of human infection with H7N9 in a city appeared to decline substantially at the same time LPMs were closed in that city for reasons other than the closure of LPMs or by chance. Information about the prevalence of H7N9 virus in poultry in different markets and the distribution network of poultry farms and markets would support construction of more complex models of the underlying transmission dynamics. Another limitation was that we could not include interventions used in locations where LPMs were not closed. Our analysis was designed to be self-controlled by comparing incidence of human cases before and after market closures. Finally, we could not examine the effect of LPM closures in other locations that had few cases during winter 2013–14. In general, our findings apply to urban areas, where live poultry purchases mainly occur in LPMs.

## Conclusions

Closure of live poultry markets was highly effective in reducing human risk for H7N9 infection during winter months of the 2013–14 influenza season. However, preventive actions such as enhanced surveillance of poultry and scheduling regular rest days could prevent the necessity of using this costly intervention.

Technical Appendix Analytic methods and procedures are discussed in this statistical analysis of the impact of live poultry market closures on influenza A(H7N9) virus transmission to humans.
