# Supplementary material for: Poultry Market Closures and Human Infection with Influenza A(H7N9) Virus, China, 2013–14
Source: Emerg Infect Dis. 2014 Nov;20(11):1891–4. doi: 10.3201/eid2011.140556 (PMC4214308; doi:10.3201/eid2011.140556)
Supplement: Technical Appendix — Analytic methods and procedures are discussed in this statistical analysis of the impact of live poultry market closures on influenza A(H7N9) virus transmission to humans. [file 14-0556-Techapp-s1.pdf]

DOI: http://dx.doi.org/10.3201/eid2011.140556

Poultry Market Closures and Human Infection with Influenza A(H7N9) Virus, China, 2013–14

Technical Appendix

Statistical Analysis of the Impact of Live Poultry Market Closures on Influenza A(H7N9) Virus Transmission to Humans

We studied the incidence rates of laboratory-confirmed human infection with influenza A(H7N9) virus in selected areas of mainland China in winter 2013-14.

In our analysis we assumed: (1) that the incidence rate of human infection with H7N9 was constant for the 2 weeks prior to the implementation of LPM closure in that area; (2) that the incidence rate of human infection with H7N9 during the LPM closure period was constant with a different rate, so that the ratio of incidence rates during versus prior to closure indicates the impact of LPM closure, with an incidence rate ratio <1 indicating a reduction in incidence; and (3) that illness onset in each human case occurred after an incubation period based on a stochastic incubation period distribution. We further assumed that the incubation periods of human cases in all cities followed the same lognormal distribution. The start date of the study time horizon for a given area was either 14 days before the start date of LPM closure or the onset date of the first confirmed local H7N9 case in 2014, whichever was later. The end date of the study time horizon was either the last day of local LPM closure or 7 March 2014, whichever was earlier, to allow for 2-3 weeks possible delay in case notification.

First, we defined the following:

N_i is the population size of area i.

$C_{i,}$ is the date of closure of live poultry market in area i.

S_i and T_i are the start and end of study time horizon for area i.

$X_{t,i}$ is the number of confirmed cases with onset on day t in area i.

We assumed that the population in area i was subject to a daily per capita force of infection $\pi_{pre,i}/N_i$ before any live poultry market (LPM) was closed and $\pi_{post,i}/N_i$ after all LPMs were closed. New infections in area i occurred according to a Poisson process such that the number of infections on day t was Poisson distributed with mean $\lambda_{pre,i} = p_i\pi_{pre,i}$ for $t \in [S_i, C_{i,j} - 1]$ and $\lambda_{post,i} = p_i\pi_{post,i}$ for $t \in [C_i, T]$ where p_i was the ascertainment proportion associated with the confirmed cases in area i. We assumed that the incubation period followed the same (cumulative) probability distribution F with mean μ and coefficient of variation c for all areas. Under these assumptions, the number of cases with onset on day t in area i was Poisson distributed with mean

$$y_{t,i,j} = \begin{cases} \lambda_{pre,i} & \text{for } S_i \le t \le C_i - 1 \\ \lambda_{pre,i}\left(1 - F(t)\right) + \lambda_{post,i}F(t) & \text{for } C_i \le t \le T_i \end{cases}$$

We estimated the pre- and post-LPM closure and the mean incubation time μ by fitting the model to the epidemic curve data in the nine areas using Markov Chain Monte Carlo methods (http://www.lce.hut.fi/teaching/S-114.202/k98/mcmc_prac.html). We assumed that F follows a lognormal distribution. The priors of the parameters for F were based on our previous study of the effect of LPM closure on H7N9 incidence (*1*):

The prior for μ was a lognormal distribution with mean 3.3 days and 97.5th percentile 5.7 days. The prior for c was a lognormal distribution with mean 0.76 and 97.5th percentile 5.2.

We assumed non-informative flat priors for all other parameters. We implemented Monte Carlo Markov Chain (MCMC) using the Metropolis algorithm to obtain posterior distributions of the parameters. A random step size was chosen for each parameter at every iteration and the variance of the step size for each parameter was automatically adjusted such that the acceptance proportion was between 30% and 70% for each parameter. The MCMC was run for 500,000 iterations for each parameter and the posterior distributions were compiled from the final 70% of the iterations.

Reference

1. Yu H, Wu JT, Cowling BJ, Liao Q, Fang VJ, Zhou S, et al. Effect of closure of live poultry markets on poultry-to-person transmission of avian influenza A H7N9 virus: an ecological study. Lancet. 2014;383:541–8. PubMed http://dx.doi.org/10.1016/S0140-6736(13)61904-2
